# S-acylation dependent post-translational cross-talk regulates large conductance calcium- and voltage- activated potassium (BK) channels

**DOI:** 10.3389/fphys.2014.00281

**Published:** 2014-08-05

**Authors:** Michael J. Shipston

**Affiliations:** Centre for Integrative Physiology, College of Medicine and Veterinary Medicine, University of EdinburghEdinburgh, UK

**Keywords:** acylation, palmitoylation, phosphorylation, trafficking, *KCNMA1*, *KCNMB4*, MaxiK channel, Slo1

## Abstract

Mechanisms that control surface expression and/or activity of large conductance calcium-activated potassium (BK) channels are important determinants of their (patho)physiological function. Indeed, BK channel dysfunction is associated with major human disorders ranging from epilepsy to hypertension and obesity. S-acylation (S-palmitoylation) represents a major reversible, post-translational modification controlling the properties and function of many proteins including ion channels. Recent evidence reveals that both pore-forming and regulatory subunits of BK channels are S-acylated and control channel trafficking and regulation by AGC-family protein kinases. The pore-forming α-subunit is S-acylated at two distinct sites within the N- and C-terminus, each site being regulated by different palmitoyl acyl transferases (zDHHCs) and acyl thioesterases (APTs). S-acylation of the N-terminus controls channel trafficking and surface expression whereas S-acylation of the C-terminal domain determines regulation of channel activity by AGC-family protein kinases. S-acylation of the regulatory β4-subunit controls ER exit and surface expression of BK channels but does not affect ion channel kinetics at the plasma membrane. Furthermore, a significant number of previously identified BK-channel interacting proteins have been shown, or are predicted to be, S-acylated. Thus, the BK channel multi-molecular signaling complex may be dynamically regulated by this fundamental post-translational modification and thus S-acylation likely represents an important determinant of BK channel physiology in health and disease.

## Introduction

The pore-forming α-subunits of large conductance calcium- and voltage- activated potassium (BK) channels are encoded by only a single gene, *KCNMA1*, yet these channels display considerable functional diversity to control an eclectic array of physiological processes in distinct cells and systems of the body (Salkoff et al., 2006; Contreras et al., 2013). Multiple mechanisms exist and work combinatorially to expand this physiological diversity including alternative pre-mRNA splicing of the α-subunit (Fodor and Aldrich, 2009), assembly with regulatory and accessory β- and γ- subunits (Orio et al., 2002; Yan and Aldrich, 2012) and post-translational modification via a diverse array of signaling pathways (Schubert and Nelson, 2001; Hou et al., 2009; Toro et al., 2013). These mechanisms ultimately control either the number of BK channels that are resident at a plasma membrane or affect the intrinsic properties or regulation of the channel at the membrane. For BK channels, due to their large conductance, small changes in either the number or activity of the channel can dramatically modify potassium flux across the membrane with subsequent impact on cellular physiology. Indeed, disruption of BK channel function is linked to a variety of disorders of the vascular, nervous, endocrine and renal systems including hypertension, obesity, epilepsy, autism, incontinence and stress-related disorders (Brenner et al., 2000, 2005; Meredith et al., 2004; Sausbier et al., 2004, 2005; Du et al., 2005; Werner et al., 2005; Jiao et al., 2011; Deng et al., 2013).

**Figure 1:**
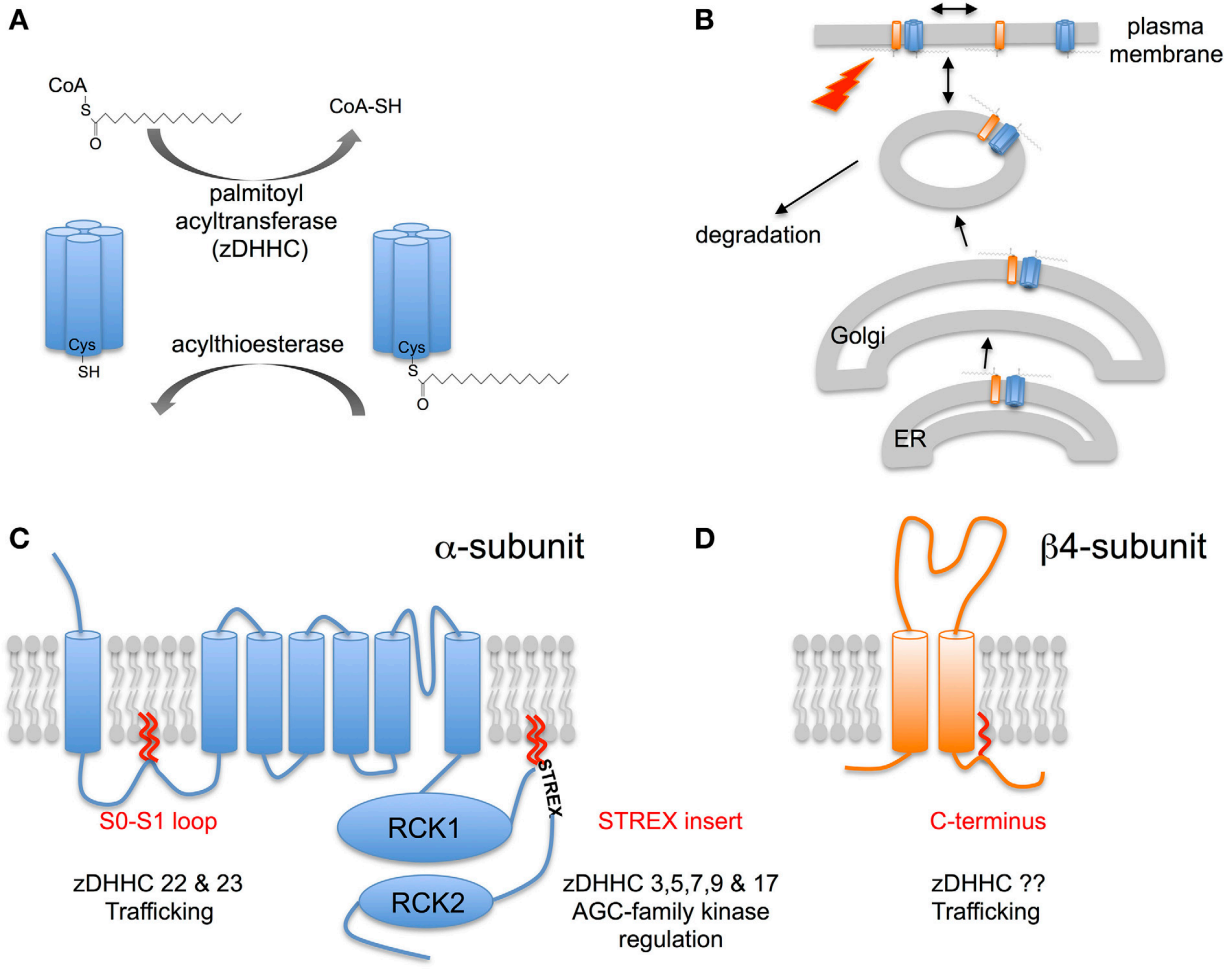
**S-acylation of BK channels. (A)** Schematic of reversible enzymatic regulation S-acylation of proteins. Addition of lipid (typically palmitate) to cysteine residues in target proteins via a thioester bond is catalyzed by a family of palmitoyl acyltransferases (zDHHCs). Removal of lipid results from the action of acylthioesterases. **(B)** S-acylation controls multiple steps in the lifecycle of BK channels including control of forward trafficking, surface expression and intrinsic channel properties and modulation by other signaling pathways. **(C)** Schematic of the pore-forming α-subunit of the BK channel encoded by the single *KCNMA1* gene. α-subunits are S-acylated at two distinct sites by distinct acyl transferase (zDHHCs): the conserved intracellular S0-S1 loop and the alternatively spliced STREX insert in the C-terminal linker between the two regulator of potassium conductance (RCK) domains. S-acylation of the S0-S1 loop controls surface trafficking of the channel whereas S-acylation of the STREX insert determines channel activity and regulation by AGC-family protein kinases. **(D)** Schematic of the regulatory β4-subunit encoded by the *KCNMB4* gene. The β4-subunit is S-acylated at a single cysteine juxtaposed to the second transmembrane domain in the intracellular C-terminus. S-acylation of the β4-subunit controls surface expression of distinct BK channel α-subunit splice variants.

Thus, post-translational modifications that control BK channel surface expression and/or activity represent powerful mechanisms to regulate both the normal physiological function of BK channels as well as serve as potential nodes of channel disruption in disease. In the past few years S-acylation, the only true fully reversible post-translational lipid modification of proteins (Figure 1A), has emerged as a fundamental mechanism controlling the surface expression and activity of a diverse array of ion channels, including BK channels (Shipston, 2011, 2014). Although S-acylation was first described more than 30 years ago, at the same time as tyrosine kinase phosphorylation, only relatively recently have the enzymes that control S-acylation been identified (Fukata et al., 2004; Linder and Deschenes, 2007; Fukata and Fukata, 2010; Greaves and Chamberlain, 2011; Resh, 2012) and an array of proteomic and imaging tools developed (Drisdel and Green, 2004; Forrester et al., 2009; Hannoush and Sun, 2010; Martin et al., 2012) to allow rigorous examination of the functional role of protein S-acylation.

As well as providing new insights into BK channel regulation and physiology these studies are also revealing important mechanisms, properties, and function of protein S-acylation.

## S-acylation: a reversible lipid post-translational modification

Protein S-acylation involves the post-translational addition of a lipid (typically, but not exclusively, palmitate) via a labile thioester bond to intracellular cysteine residues. Thus, S-acylation, unlike other lipid modifications such as myristoylation or prenylation, is dynamically reversible (Figure 1A). Indeed, S-acylation of most proteins is enzymatically driven by a large family (23 in mammals) of transmembrane zinc-finger containing protein acyltransferases (zDHHC family). zDHHCs have a highly conserved Asp-His-His-Cys (DHHC) signature sequence within a cysteine rich stretch of ~50 amino acids critical for catalytic activity. zDHHCs display distinct tissue expression as well as subcellular localization with zDHHCs expressed typically at the ER, Golgi or plasma membrane (Fukata et al., 2004; Linder and Deschenes, 2007; Fukata and Fukata, 2010; Greaves and Chamberlain, 2011; Resh, 2012). Conversely, de-acylation is determined by acylthioesterases belonging to the large serine hydrolase superfamily including the cytosolic LYPLA1, LYPLA2 enzymes, and the lysozomal PPT1 (Zeidman et al., 2009; Bachovchin et al., 2010; Martin et al., 2012). For most proteins, let alone ion channels, the zDHHCs or thioesterases that control S-acylation are poorly characterized.

Over the last few years developments in both characterization of the enzymes that control S-acylation as well as improved bicohemical methods to interrogate native protein S-acylation have begun to reveal the a major role for S-acylation in controlling both trafficking and regulation of many different types of ion channel, incuding BK channels (Figure 1B).

## S-acylation of BK channel pore-forming α-subunits

### BK channel α-subunits are S-acylated at two distinct sites

In native tissues, such as brain, BK channels pore-forming α-subunits are robustly S-acylated as revealed using biotin-exchange or acyl-resin assisted capture (acyl-RAC) methodologies (Kang et al., 2008; Tian et al., 2008; Alioua et al., 2011). Although, these approaches did not reveal cysteine residues that are S-acylated freely available S-acylation prediction algorithms [e.g., CSS-palm (http://csspalm.biocuckoo.org) (Ren et al., 2008)] reveal that cysteine residues in both the intracellular S0-S1 loop as well as the alternatively spliced stress-regulated exon (STREX) insert, in the C-terminal linker between the two regulator of K conductance (RCK) domains, are likely S-acylated (Figure 1C). Using a combination of site directed mutagenesis, acyl-RAC and ^3^H-palmitate labeling of recombinant murine ZERO variant BK channels (that lack the STREX insert) expressed in HEK293 cells revealed that cysteine residues C53 and 56 in the S0-S1 loop were endogenously S-acylated in HEK293 cells (Jeffries et al., 2010; Tian et al., 2012). Site directed mutagenesis of C53 and C56 abolished S-acylation of the full-length ZERO variant suggesting these are the only cysteines S-acylated in the entire ZERO channel. S-acylation of the S0-S1 loop allows the isolated S0-S1 loop (assayed as a –GFP fusion protein) to associate with the plasma membrane in the absence of transmembrane domains. This suggests that S-acylation acts as an additional membrane anchor in this otherwise largely unstructured domain. Using an siRNA based screen to knock-down the expression of individual zDHHCs expressed endogenously in HEK293 cells revealed that zDHHC 22 and zDHHC 23 are the major acyltransferases that control S-acylation of the S0-S1 loop (Jeffries et al., 2010; Tian et al., 2012). Whether, these distinct zDHHCs differential control S-acylation of C53 or C56, respectively is unknown. However, the zDHHCs may also control S-acylation of the S0-S1 loop at different stages during the lifecycle of the channel as it traffics from the ER to plasma membrane (Figure 1B). Furthermore, overexpression of zDHHC23, but not the catalytically inactive zDHHS23 mutant, increased S0-S1 S-acylation.

Similar approaches have also revealed that the alternatively spliced STREX insert located within the unstructured linker between RCK1 and RCK2 in the large intracellular C-terminus is S-acylated at two tandem cysteine residues: C645 and C646 (Tian et al., 2008, 2010; Jeffries et al., 2012). For example, using an imaging screen exploiting a –GFP fusion of the entire C-terminus of the ZERO or STREX variant BK channel (i.e., in the absence of transmembrane domains) revealed that the STREX C-terminus, but not the ZERO C-terminus, was robustly associated with the plasma membrane. Site directed mutagenesis of the two cysteines, C645 and C646 in STREX predicted to be S-acylated by the CSS-palm algorithm, abolished S-acylation and membrane association of the fusion protein (Tian et al., 2008; Jeffries et al., 2012). Similar data were also obtained using a –GFP fusion of just the 59 amino acid STREX insert alone. S-acylation of the STREX insert at these residues was confirmed in full-length STREX channels in which the S0-S1 S-acylated cysteines were mutated to alanine (Tian et al., 2008; Jeffries et al., 2012). Using the siRNA screen to knockdown individual zDHHCs revealed several zDHHCs (zDHHCs 3, 5, 7, 9, and 17) as potential S-acylating enzymes of the STREX insert (Tian et al., 2010). Moreover, S-acylation of the STREX domain was enhanced by over expression of the cognate zDHHCs with zDHHC17 showing the greater selectivity for the dicysteine C645:646 motif.

S-acylation is a reversible post-translational modification catalyzed by members of the serine hydrolase superfamily. The lysosomal acylthioesterase, PPT1 that typically de-acylates proteins undergoing lysosomal degradation, appears to have little role in controlling S0-S1 loop S-acylation. In contrast, over expression of the cytosolic thioesterase LYPLA1 and a splice variant of the related LYPLAL1, but not LYPLA2, depalmitoylated BK channels at the S0-S1 loop (Tian et al., 2012). In both cases, catalytically “dead” mutants of the thioesterases were ineffective. However, steady-state S-acylation of the S0-S1 loop was not significantly affected by knockdown of LYPLA1 suggesting that either deacylation is not rate limiting or that S-acylation of the S0-S1 loop has a long half life in the lifecycle of the channel. To date, the acyltransferases controlling STREX insert S-acylation, and the dynamics of S-acylation of either site are not fully elucidated.

Taken together, these data reveal that the pore-forming α-subunits can be S-acylated at distinct intracellular domains of the channel. Remarkably, each site is differentially regulated by distinct zDHHC enzymes suggesting that each site can be controlled independently and supporting the hypothesis that distinct zDHHCs can display substrate specificity (Greaves and Chamberlain, 2011). This also raises a challenge for interrogating BK S-acylation in native tissues. For example, to interrogate how S-acylation of each domain may be differentially controlled by distinct physiological challenges will require adaptation of current biochemical assays. As an example, using the acyl-RAC approach would require approaches such as on-bead tryptic digests followed by elution of S-acylated peptides for analysis by mass spectrometry. The zDHHCs that control S0-S1 loop or STREX insert S-acylation are expressed at either the ER, Golgi or plasma membrane suggesting that the pore-forming α-subunits may be regulated at multiple sites in the trafficking pathways to the plasma membrane. Clearly, a challenge for the future is also to establish the tissue and cellular distribution of these zDHHCs and thioesterase and their regulation of BK channels in native tissues.

Importantly, as S-acylation controls two functionally distinct domains on the pore-forming α-subunits this suggests that differential S-acylation may control distinct channel properties as discussed below.

### S0-S1 loop S-acylation controls cell surface trafficking

The functional role of the BK channel S0-S1 loop is poorly understood although it is thought to be largely unstructured and includes residue(s) important for magnesium ion coordination with the C-terminal RCK domains (Yang et al., 2007; Cui, 2010; Shi et al., 2013). Furthermore, in some species it is also a site for both post-translational modification by phosphorylation as well as sites of alternative splicing (c.f. Liu et al., 2006). Moreover, the transmembrane S0 domain is important for functional coupling with regulatory β-subunits (Orio et al., 2002). Thus, potentially S-acylation of the S0-S1 loop may have multiple functional consequences.

**Figure 2:**
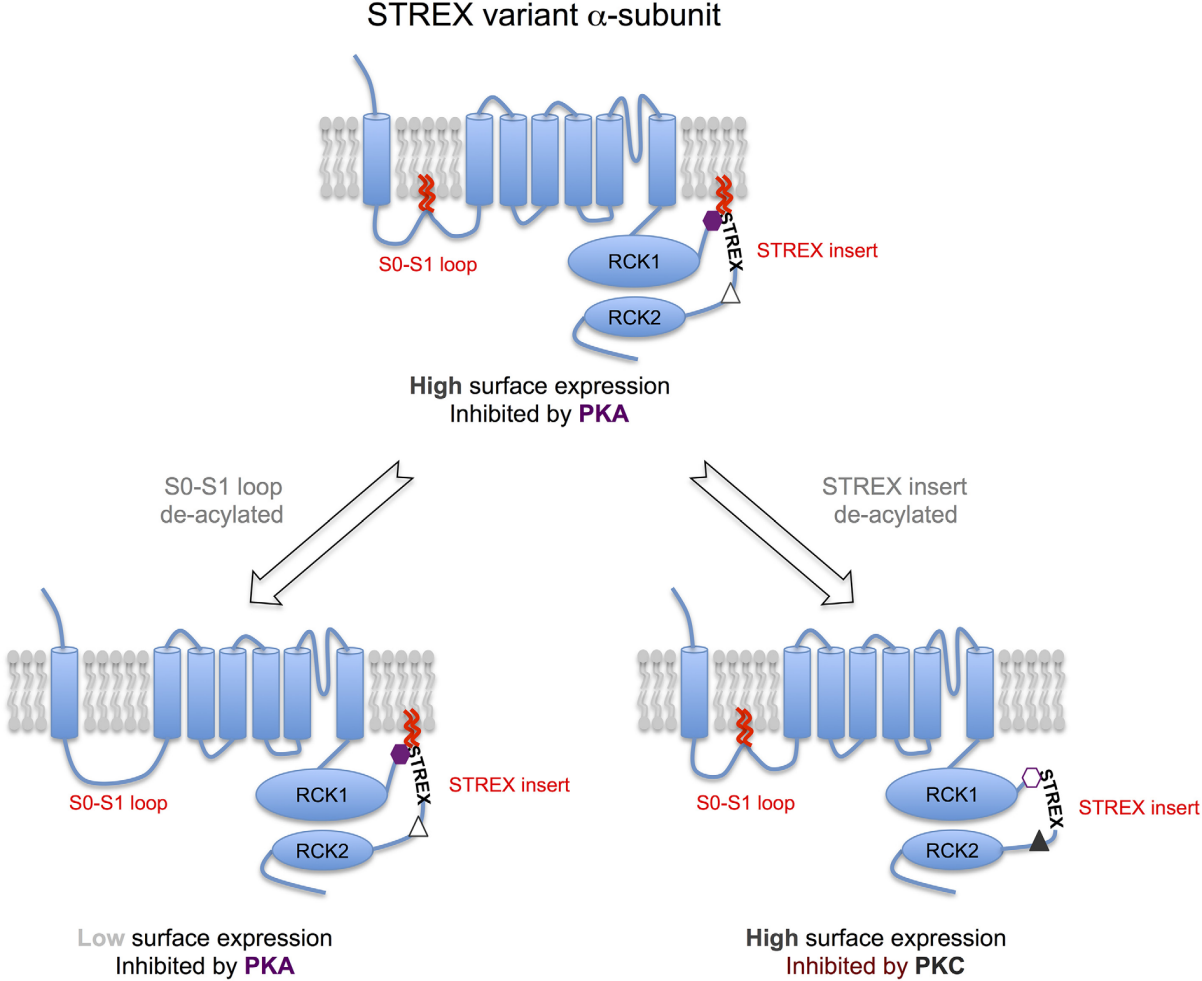
**S-acylation of the α-subunit controls distinct properties of BK channels**. Model schema for regulation of STREX variant α-subunits by S-acylation and AGC-family kinases. The STREX variant of the BK channel can be S-acylated at two distinct sites: (i) the S0-S1 loop allowing the loop to associate with the plasma membrane and is important in controlling surface delivery of the BK channel; (ii) in the alternatively spliced STREX insert that allows the cytosolic STREX domain to interact with the plasma membrane. S-acylation of the STREX insert determines STREX channel regulation by AGC-family protein kinase dependent phosphorylation. Protein kinase A (PKA)-dependent phosphorylation of S^636^ in the STREX insert (purple hexagon), that is immediately upstream of the S-acylated cysteine residues, results in dissociation of the STREX domain from the plasma membrane and inhibition of STREX channel activity. In contrast, when STREX is S-acylated, protein kinase C (PKC) has no effect on channel activity even though phosphorylation of the PKC-consensus sites (S^700^ and S^1156^), that are downstream of the STREX insert, result in channel inhibition in channels lacking the STREX insert (e.g., ZERO variant). Thus, the S-acylated STREX insert prevents PKC-mediated inhibition in STREX channels. However, deacylation of the STREX insert, or PKA mediated dissociation of the STREX domain from the plasma membrane, now allows phosphorylation of the S^700^ PKC-site (S^700^, gray triangle) that, in conjunction with the C-terminal PKC site S^1156^, confers PKC-dependent inhibition of STREX channels. Thus, the S-acylation of STREX serves as a switch to determine STREX BK channel regulation by either PKA or PKC. PKG- mediated activation of STREX channels, dependent on phosphorylation of other C-terminal serine residues, is not controlled by STREX insert S-acylation. In combination with control of S0-S1 loop S-acylation channels with distinct surface expression and regulation by AGC-kinases can be generated thus expanding BK channel physiological diversity. For example, channels that are S-acylated at both the S0-S1 loop and STREX insert (center top) would be predicted to have high surface expression and inhibited by PKA. Channels de-acylated at only the S0-S1 loop would have low surface expression but inhibited by PKA (bottom left) whereas channels S-acylated at only the STREX insert would have high surface expression and now inhibited by PKC, but not PKA (bottom right).

However, the major role of S-acylation of the S0-S1 loop appears to be in the control of cell surface expression of α-subunits (Jeffries et al., 2010; Tian et al., 2012) (Figures 1B, 2). Depalmitoylation of the S0-S1 loop, using several approaches including inhibition of global S-acylation with the broad spectrum zDHHC inhibitor 2-bromopalmitate (2-BP), siRNA mediated knockdown of zDHHCs 22 or 23, or site directed mutagenesis of the S-acylated cysteine residues to alanine, results in a suppression of BK channel steady-state surface expression by more than 50%. This effect appears to be independent of the splice variant of the α-subunit under investigation, including whether the C-terminal STREX insert is S-acylated (Jeffries et al., 2010; Tian et al., 2012; Chen et al., 2013b). Although, the suppression of surface expression can be partially rescued by expression with some regulator subunits (see section S-acylation of β4-Subunits Controls Surface Delivery and Chen et al., 2013b) de-palmitoylated α-subunits surface expression is still compromised compared to expression of S-acylated subunit and regulatory subunits. Thus, S-acylation of S0-S1 plays a dominant role in surface expression. BK channel α-subunits that can never be S-acylated at the S0-S1 loop (e.g., site directed cysteine to alanine mutants) show enhanced trapping at the Endoplasmic reticulum suggesting that ER exit may be a key regulatory step, although S-acylation is not essential as mutant α-subunits can still reach the cell surface. However, a key regulatory site for BK channel trafficking controlled by the S0-S1 loop is also at the level of exit from the trans-golgi network (TGN) (Tian et al., 2012). Overexpression of the acylthioesterase LYPLA1, but not LYPLA2, also decreased steady-state cell surface expression by approximately half in accordance with the studies above. Channels that were de-acylated by LYPLA1 were largely retained in the TGN and this TGN accumulation was associated with a reduction in channel co-localization in recycling endosomes (Tian et al., 2012). Clearly, a major goal will be to understand the spatiotemporal dynamics of S0-S1 S-acylation during both forward trafficking to the cell surface as well as routes for channel recycling.

As indicated above, channels that are de-acylated can still reach the plasma membrane. Recent studies that S0-S1 loop S-acylation (using site directed alanine mutants) controls the lateral mobility of single BK channels in the plasma membrane (Kim et al., 2014). De-acylated channels display a faster and more random lateral diffusion compared to wild-type channels suggesting S-acylation constrains the movement of channels in the plasma membrane (Kim et al., 2014). Whether this reduced mobility is a function of the ability of the S-acylated S0-S1 loop to act as an additional membrane anchor or allows BK channels to assemble with the cytoskeleton, or other membrane domain organizing components, remains to be determined.

S0-S1 loop S-acylation does not have any significant effect on the intrinsic calcium/voltage sensitivity of the α-subunit as site directed mutation of the S-acylated cysteine residues has no effect on the voltage for half maximal activation over a range of calcium concentrations (Jeffries et al., 2010; Kim et al., 2014). This also suggests that S-acylation is not central to the role of the S0-S1 loop in coordinating magnesium binding with the RCK domains. However, whether S0-S1 loop S-acylation controls physical and/or functional coupling to regulatory β- or γ- subunits remains to be determined.

### S-acylation of a RCK1-RCK2 linker splice variant (STREX) determines regulation by AGC family protein kinases

BK channels are subject to post-translational regulation by an array of distinct protein kinases and phosphatases including members of the classical AGC-family of protein kinases: cAMP dependent protein kinase (PKA); protein kinase C (PKC) and cGMP-dependent protein kinase (PKG). Importantly, the effect of AGC-family kinase mediated phosphorylation on the activity of BK channels is determined by the pore-forming α-subunit splice variant (e.g., Tian et al., 2001, 2004; Zhou, 2001). In this regard, inclusion of the alternatively spliced STREX insert introduces an additional consensus PKA-phosphorylation that switches channel regulation by PKA from channels that are activated (when the STREX insert is absent) to channels that are inhibited (when the STREX insert is included) (Tian et al., 2001, 2004). The STREX insert is a cysteine rich domain that is located in the structurally disordered intracellular linker between the RCK1 and RCK2 domains in the C-terminus (Figure 1C). As well as controlling regulation by PKA, the STREX insert also shifts the voltage for half-maximal activation to the left such that STREX BK channels are more sensitive to calcium and voltages in the physiological range and also changes both activation and deactivation kinetics (Xie and McCobb, 1998; Tian et al., 2001). Intriguingly, the PKA consensus site required for PKA-mediated inhibition within STREX (S636) is located immediately upstream of the di-cysteine cluster (C645:646) that is S-acylated, and within a highly polybasic sequence of amino acids (Figure 1C). As outlined above (section BK Channel α-Subunits are S-acylated at Two Distinct Sites) the S-acylated STREX domain associates with the plasma membrane. PKA-mediated phosphorylation of S636, or phosphomimetic mutation to S636E or S636D, all of which introduce negative charge into the otherwise polybasic region upstream of the S-acylated dicysteines S645:646 attenuate STREX S-acylation and results in dissociation of the STREX domain from the plasma membrane (Tian et al., 2008; Jeffries et al., 2012). This data also suggest that both the polybasic domain and S-acylated di-cysteine motif are required for association of the STREX domain with the plasma membrane. Indeed, mutation of residues of the polybasic domain *per se* control S645:646 S-acylation and membrane association of STREX. As S-acylation is mediated at membrane interfaces this may suggest that the polybasic domain is required for an initial transient association with the membrane to allow S-acylation of C645:646 by cognate zDHHCs, as reported for other proteins with such polybasic domains (Linder and Deschenes, 2007; Greaves and Chamberlain, 2011). In addition, destabilization of the polybasic domain by PKA-phosphorylation may also allow cytosolic acylthioesterases access to the di-cysteine cysteines to promote de-acylation. In this way, palmitoylation and phosphorylation may interact and allow temporal control of STREX channel function.

Thus, this data supports a model in which introduction of negative charge disrupts interaction of the polybasic region upstream of the site of S-acylation, leading to conformational rearrangements in the channel and subsequent inhibition of channel properties (Figures 1C, 2). In support of this, de-acylation of STREX results in channels with kinetic and conductance/voltage relationships similar to channels inhibited by PKA, or lacking the STREX insert. Thus, pharmacological inhibition of STREX S-acylation, or knockdown of the cognate zDHHCs (as in section BK Channel α-Subunits are S-acylated at Two Distinct Sites), prevents PKA-mediated inhibition and converts STREX BK channels to a phenotype closer to that of channels lacking the STREX insert. This suggests that STREX insert S-acylation is critical for conferring core properties, as well as PKA-mediated inhibition, of STREX variant BK channels. Importantly, other modes of regulation conferred by the STREX insert, such as regulation by low oxygen tension (hypoxia) (McCartney et al., 2005) are not dependent upon S-acylation of the STREX domain. Furthermore, at least in recombinant systems, STREX insert S-acylation has no effect on plasma membrane surface expression of BK channels illustrating that S-acylation of the STREX and S0-S1 domains have distinct functions, controlled by different zDHHCs. Taken together this suggests an important level of cross-talk between the PKA and S-acylation signaling pathways via mutual control of an S-acylated domain of proteins (Figures 1C, 2)—such cross-talk is an emerging concept in a range of other ion channels and signaling proteins (Shipston, 2014).

The studies discussed above reveal a level of interaction between a phosphorylation (PKA) and lipid post-translational modification through mutual regulation of a protein domain at the plasma membrane with the phosphorylation and S-acylation sites close together (in linear sequence ~10 amino acids). However, recent studies from Thomas Wieland's laboratory examining PKC-mediated regulation of BK channel activity have also revealed a role for STREX S-acylation in controlling PKC phosphorylation mediated regulation via consensus phosphorylation sites outwith the STREX domain (Zhou et al., 2012). PKC has been shown to inhibit BK channels, in splice variants lacking the STREX insert, through phosphorylation of two distinct PKC consensus sites located in the BK channel C-terminus (Zhou et al., 2010). One PKC site is located toward the very C-terminus of the channel (S1156) with an additional site (S700) located downstream of the STREX insert site of splicing. Importantly, for PKC-mediated inhibition the S700 site is only phosphorylated once S1156 is phosphorylated and both sites must be phosphorylated for PKC to inhibit channel activity. However, in channels in which the STREX insert is included, PKC has no effect on channel activity even though both the S700 and S1156 sites are present in the C-terminus (Zhou et al., 2012). Strikingly, in STREX channels in which S-acylation of the STREX di-cysteine S-acylation motif was prevented, either by global zDHHC inhibition by 2-BP, or site directed mutagenesis of C645:646, PKC could now inhibit channel activity. Furthermore, in S-acylated STREX channels in which the S700 PKC consensus site was mutated to the phosphomimetic S700E PKC could now inhibit channel activity (Zhou et al., 2012). This suggests a model in which the S-acylated STREX domain normally occludes phosphorylation of S700 thus preventing PKC mediated inhibition. Dissociation of the STREX domain from the plasma membrane via either de-acylation *per se* or prior PKA-mediated phosphorylation of the STREX insert at the PKA site S636, would thus gate the ability of STREX variant channels to be inhibited by PKC (Figure 2). Indeed, this appears to be an important mode of regulation in pituitary endocrine cells that express STREX variant channels (Zhou et al., 2012).

In contrast, PKG mediated regulation of STREX containing, or STREX-less channels, is independent of the S-acylation status of the STREX insert *per se* (Zhou et al., 2012), most likely as the major consensus site for PKG-mediated regulation is toward the very C-terminus of the channel α-subunit. Thus, these data reveal an important role in S-acylation in acting as a switch to specify STREX variant regulation by PKA and PKC (Figure 2). Furthermore, it may also provide a mechanism to provide robust regulation by both PKA and PKC in STREX-expressing cells in which BK channels are targets for signaling pathways activating the PKC and PKA pathways such as in some endocrine cells.

## S-acylation of BK channel regulatory subunits and associated proteins

### S-acylation of β4-subunits controls surface delivery

The assembly of BK channel pore-forming α-subunits with a diverse family of transmembrane β- and γ- subunits provides additional mechanisms to determine physiological diversity of the BK channel in different cells and tissues. Regulatory subunits play diverse roles in modifying both the intrinsic properties of the channel (kinetics and calcium/voltage sensitivity) as well as trafficking of the channel to the cell surface (Orio et al., 2002; Yan and Aldrich, 2012). Recent evidence reveals that S-acylation of the regulatory β4-subunit may play an important role in the latter. The β4-subunit is highly expressed in the nervous system, as well as endocrine tissues, and confers complex effects on channel activity dependent upon the local calcium concentration and also modifies BK channel pharmacology by making the channel largely resistant to the toxins Iberiotoxin and Charybdotoxin (Meera et al., 2000; Brenner et al., 2005; Wang et al., 2006). The β4-subunit, as for other β-subunits, has two transmembrane domains with a large extracellular loop and short intracellular N- and C-termini (Figure 1D). Previous data have revealed that the β4 C-terminus contains a basic ER-retention trafficking motif that controls β4-subunit surface expression (Shruti et al., 2012). β4-subunits are S-acylated in mouse brain and when expressed in recombinant systems at a single cysteine residue (C193) juxtaposed to the second transmembrane domain immediately upstream of the C-terminal trafficking motif (Chen et al., 2013b) (Figure 1D). β4-subunits alone are typically trafficking incompetent largely residing in the ER and S-acylation has no effect on β4-subunit localization. However, mutation of the ER retention motif allows β4-subunts to traffic to the cell surface and the exit from the ER is dependent upon S-acylation of the β4-subunit at C193. Importantly, assembly of β4-subunits with α-subunits controls surface expression of the channel complex. Rather surprisingly, β4-subunits can up- or down- regulate α-subunit surface expression depending on the specific splice variant of the pore forming subunit (Shruti et al., 2012; Chen et al., 2013a). Surface expression of BK channel α-subunits that include the C-terminal splice variant..REVEDEC motif is upregulated by β4-subunits (Chen et al., 2013b). Upregulation of α-subunit surface expression is dependent upon S-acylation of the β4-subunit at C193. β4-subunits that are de-acylated at C193 do not promote surface expression of the..REVEDEC α-subunit variants. The REVEDEC heptapeptide has been reported to suppress α-subunit surface expression and thus hypothesized to act as a trafficking motif (Kim et al., 2007; Chiu et al., 2010). Transplanting the REVEDEC heptapeptide onto the C-terminus of α-subunits whose surface expression was not normally enhanced upon co-expression with β4-subunits, resulted in a β4-subunit S-acylation dependent up regulation of surface expression. This suggests that..REVEDEC acts as a trafficking motif and that S-acylated β4-subunits may mask this motif to allow enhanced surface expression. The mechanistic basis for this effect remains to be resolved however a possible explanation is that S-acylation of β4-subunits is required for the correct structural interaction with α-subunits at the ER. Chemical crosslinking experiments reveal that the extracellular aspect of the second transmembrane domain of the β4-subunit is in close apposition to S0 of the α-subunit (Wu et al., 2009). In other systems, S-acylated cysteines juxtaposed to transmembrane domains promote tilting of the transmembrane domain and this may be important at the thinner ER membrane to reduce hydrophobic mismatch as well as confer restraints on the β4-peptide (Nyholm et al., 2007; Abrami et al., 2008; Baekkeskov and Kanaani, 2009; Charollais and der Goot, 2009).

However, although such a model may explain how β4-subunits control surface expression of the α-subunit..REVEDEC splice variant, β4-subunit control of BK channel surface expression is clearly more complex than the model outlined above. Indeed, surface expression of another distinct α-subunit splice variant was in fact suppressed upon co-expression with *de*-acylated β4-subunits. This is in accordance with other data supporting a role for β4-subunits to suppress BK channel expression in some neurons, although how selectivity of action between different α-subunit splice variants is conferred is not known. Moreover, although β4-subunits can also enhance surface expression of de-acylated α-subunit..REVEDEC splice variants at the S0-S1 loop, an effect that is β4-subunit S-acylation dependent, β4-subunit co-expression cannot rescue to the levels achieved by expression of α- and β4-subunits that can both be S-acylated. Thus, in BK channels, S-acylation of the S0-S1 loop of the pore-forming subunit controls global BK channel surface expression and β4-subunit S-acylation additionally controls surface expression of specific pore-forming subunit splice variants.

### Control of BK channels via S-acylation of other components of the channel complex?

BK channels, as for other ion channels, do not exist in “isolation” in membranes but assemble into functional complexes with an array of adapter, structural and signaling proteins (e.g., see Lu et al., 2006; Kathiresan et al., 2009; Berkefeld et al., 2010; Toro et al., 2013). As S-acylation can control a wide variety of proteins, from G-protein coupled receptors, to tyrosine kinases and multifunctional adapter proteins such as PSD-95 (Fukata et al., 2004; Linder and Deschenes, 2007; Fukata and Fukata, 2010; Greaves and Chamberlain, 2011; Shipston, 2011, 2013; Resh, 2012), S-acylation may also exert effects on BK channel trafficking and function through modulation of the channel multi-molecular complex *per se* or signaling pathways that converge on the complex. As assembly of BK channels with different interacting proteins is likely to be cell specific S-acylation may control BK channel function differentially through modulation of components of the larger multi-molecular BK channel signaling complex.

**Figure 3:**
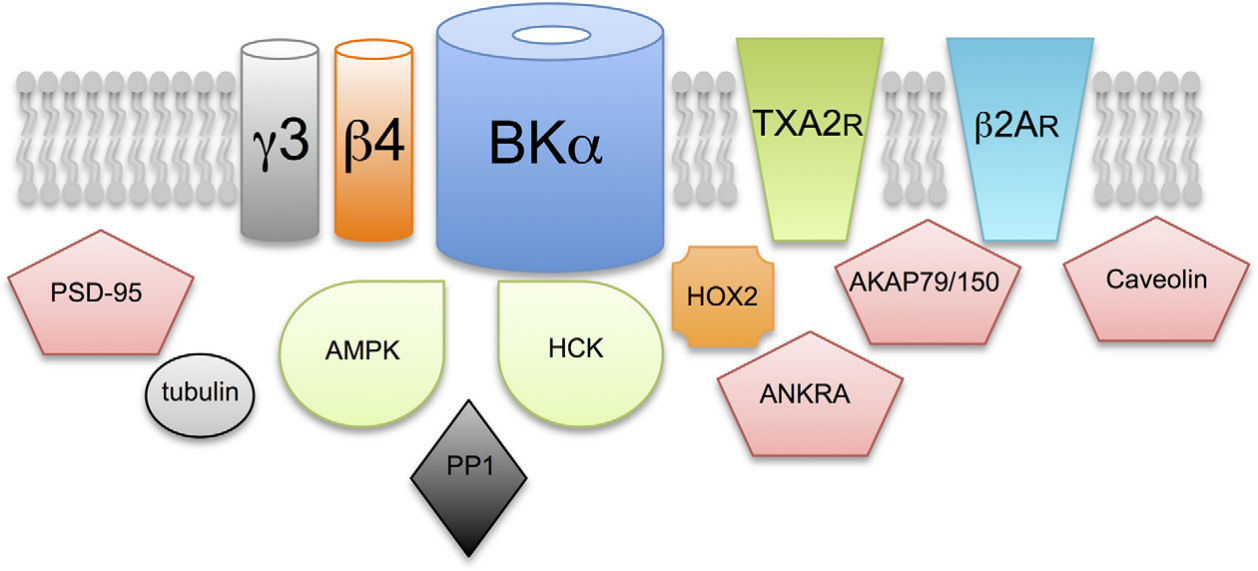
**Does S-acylation control multiple components of the BK channel signaling complex?** Schematic illustrating exemplar proteins that have previously been reported to assembly within a multimolecular complex with the BK channel pore forming α-subunit and are strongly predicted, or have been previously shown, to be S-acylated in cells. The functional consequence of S-acylation, except for the β4-regulatory subunit, of these components on BK channel function is not yet known. Proteins include: additional accessory subunits of the BK channels such as the γ3 subunit LRRC55; G-protein coupled receptors such as the thromboxane A2 (TXA2R) and β2-adrenergic (β2AR) receptors; adapter and scaffolding proteins such as PSD-95, AKAP79/150, caveolin and ANKRA; structural and cytoskeletal proteins such as tubulin and signaling proteins such as the AMP-activated serine/threonine protein kinase (AMPK), the tyrosine kinase, HCK and the serine/threonine protein phosphatase PP1.

As outlined in section S-acylation of β4-subunits Controls Surface Delivery β4-subunits are S-acylated and control trafficking however, although other regulatotry subunits are also predicted to be S-acylated (Figure 3) the functional consequence is as yet unknown. Furthermore, a significant number of BK channel interacting proteins previously reported in proteomic screens (e.g., see Lu et al., 2006; Kathiresan et al., 2009; Berkefeld et al., 2010; Toro et al., 2013) are also predicted, or have been shown, to be S-acylated (Figure 3). A clear challenge will be to define S-acylated components of the BK channel complex in defined cell types and understand both how S-acylation of these proteins is controlled as well as the functional consequence of S-acylation on BK channel physiology.

## Conclusions

Multiple mechanisms, including alternative splicing, assembly with accessory subunits and post-translational modifications allow considerable functional diversity of BK channels to be generated from a single gene, *KCNMA1*, encoding for the pore-forming α-subunits. The work described above implicates S-acylation, a reversible post-translational lipid modification of proteins, as a major mechanism to control both the number of BK channels at the cell surface as well as their activity and regulation at the membrane. Importantly, S-acylation can control multiple sites within the BK channel complex and this post-translational modification works combinatorially with other mechanisms that specify functional diversity to fine tune BK channel properties and regulation.

Clearly to understand the contribution of this important post-translational modification in BK channel physiology several major challenges need to be addressed. Firstly, although we are beginning to define the enzymes that control BK channel S-acylation the temporal and spatial dynamics of BK channel S-acylation is very poorly understood. Furthermore, although distinct enzymes can control different aspects of BK channel physiology how these enzymes themselves are regulated is largely unknown. As S-acylation works in combination with other mechanisms, including controlling cysteine reactivity *per se* (Hess et al., 1993; Sen and Snyder, 2010; Ho et al., 2011), a clear challenge is to define how such interactions control specific cellular functions. For example, defining cellular physiological processes and specific cell types in which S-acylation controls: AGC-family kinase regulation of the STREX variant, β4-subunit mediated regulation of α-subunit splice variant surface expression, or regulation conferred by assembly with other S-acylated components of the BK channel complex. Ultimately the major challenge will be to understand how S-acylation controls BK channel function at the systems and whole animal level and understanding how palmitoylation status may be controlled and/or dysregulated in disease. For example, several of the zDHHC enzymes implicated in BK channel control, such as zDHHCs 5, 9, and 17, are also implicated in a variety of disorders including endocrine dysfunction and neurological deficits. In contrast, the physiology of other zDHHC enzymes that control BK channel function, such as zDHHC23, is completely unexplored.

With the continued development of new tools to interrogate and manipulate S-acylation we are now at the foothills of being able to define and understand the role of S-acylation in the physiological function of BK channels as well as providing an opportunity to gain greater insights into the mechanisms and function of S-acylation itself.

### Conflict of interest statement

The author declares that the research was conducted in the absence of any commercial or financial relationships that could be construed as a potential conflict of interest.
